# Efficacy of Pregabalin in Childhood Refractory Partial Seizure

**Published:** 2014-01-26

**Authors:** Gholamreza Zamani, Alireza Tavasoli, Ameneh Zare-Shahabadi, Nima Rezaei, Alireza Ahmadvand

**Affiliations:** 1 Department of Pediatric Neurology; 2 Research Center for Immunodeficiencies, Pediatrics Center of Excellence, Children's Medical Center; 3 Department of Immunology, Molecular Immunology Research Center; 4 Department of Health, Tehran University of Medical Sciences, Tehran, Iran

**Keywords:** Pregabalin, Clinical trial, Refractory Partial Seizure, Seizure, Children

## Abstract

***Objective:*** About one third of partial seizures are refractory to treatment. Several anticonvulsant drugs have entered the market in recent decades but concerns about intolerance, drug interactions, and the safety of the drug are notable. One of these new anticonvulsants is pregabalin, a safe drug with almost no interaction with other antiepileptic drugs.

***Methods:*** In this open label clinical trial study, pregabalin was used for evaluation of its efficacy on reducing seizure frequency in 29 children suffering from refractory partial seizures.

Average daily and weekly seizure frequency of the patients was recorded during a 6-week period (baseline period). Then, during a period of 2 weeks (titration period), pregabalin was started with a dose of 25-75 mg/d, using method of flexible dose, and was brought to maximum dose of drug that was intended in this study (450 mg/d) based on clinical response of the patients and seizure frequency. Then the patients were given the drug for 12 weeks and the average frequency of daily and weekly seizures were recorded again (treatment period).

***Findings*** ***:*** Reduction in seizure frequency in this study was 36% and the responder rate or number of patients who gained more than 50% reduction in seizure frequency was 51.7%.

***Conclusion:*** This study showed that pregabalin can be used with safety and an acceptable efficacy in treatment of childhood refractory partial seizures.

## Introduction

Epilepsy is one of the most common chronic neurologic disorders, affecting an estimated 50 million people worldwide (or about 1-2% of population). A significant proportion of neurologic disabilities is related to epilepsy and its associated conditions. Partial seizures are seen more frequently than generalized type in both pediatric and adult populations^[^^1^^]^. 50% of children with partial seizures are controlled with first medication. Using a second anti-epileptic drug, the success rate reaches about 60-70%. So about 30% of partial seizures, are difficult to control (refractory epilepsies)^[^^2^^,^^3^^]^. In selected patients, surgical treatment may be curative but overall, there is no unique strategy for treatment of all epileptic patients, and yet medical treatment with anti-epileptic drugs is the main focus of therapeutic plans^[^^4^^,^^5^^]^. Drug-resistant epilepsy is associated with cognitive and behavioral problems and impaired psychosocial development in addition to increasing risk of injury and even death caused by recurrent seizures^[^^6^^]^. 

**Fig. 1:**
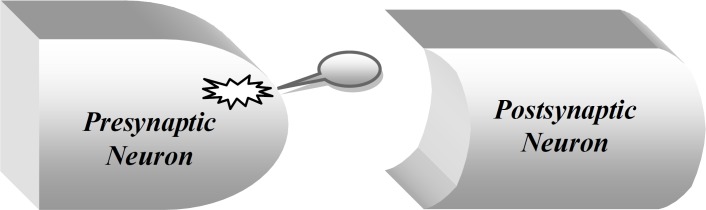
Schematic presentation of pregabalin action on voltage-gated calcium channels in presynaptic neuron(۞)

 The management of epilepsy should be towards complete control of seizures with respect to minimizing the occurrence of adverse effects of drugs and improving the patient’s quality of life^[^^4^^]^. Pregabalin (Lyrica™) is one of the latest additions in the antiepileptic medication regimen that is structurally similar to Gabapentin. It was approved by Food and Drug Administration in USA, 2005, as an add-on therapy for partial epilepsy, post-herpetic neuralgia, and fibromyalgia. Controlled clinical trials demonstrated its effectiveness on peripheral and central neuropathic pain, and in the treatment of generalized anxiety disorder^[^^4^^-^^7^^]^. This drug crosses the blood-brain barrier, and binds potently to the α_2_-d subunit, an auxiliary protein associated with voltage-gated calcium channels in the central nervous system, attenuating depolarization-induced Ca^2+^ influx in nerve terminals that results in decreasing the level of the excitatory neurotransmitter, glutamate, noradrenaline and substance P (Fig. 1)^[^^2^^,^^6^^]^.

 Its pharmacological and pharmacokinetic profiles such as highly predictable and linear pharmacokinetics across the dose range (150–600 mg/day) with low inter-subject variability, rapid and extensive absorption following oral dosing with peak plasma concentrations occurring approximately 1 h after oral intake and steady state being achieved within 24–48 h following repeated administration, provide a consistent and predictable basis for its use in clinical practice as an add-on antiepileptic agent^[^^2^^]^. This 12-week, open label study adds to the accumulating evidence of the efficacy and safety of pregabalin as an adjunctive treatment for refractory partial seizures^[^^8^^-^^13^^]^. No regional data evaluating the use of pregabalin in Iranian epileptic patients have been published so far. Herein, the results of a clinical trial on usage of pregabalin for the treatment of children with severe drug-resistant partial epilepsy are presented.

## Subjects and Methods

**Study Design**

This 12-week, open-labeled, before-after study of pregabalin as adjunctive treatment for refractory partial seizures was conducted from June 2011 to March 2012, in the Children's Medical Center Hospital, Pediatrics Center of Excellence, Tehran. The study protocol was approved by the Institutional Review Board. All patients provided written informed consent before entering the study. This study was conducted in compliance with the Declaration of Helsinki and the International Conference on Harmonization Good Clinical Practice Guidelines. This study was also registered in the Iranian Registry of Clinical Trials. The study comprised three main phases; after selecting the patients and before the onset treatment, in a period of 6 weeks (baseline phase), average daily and weekly seizures were recorded by the patient, a trained observer or a legal guardian in a diary pad. After that, during a period of 2 weeks (dose-optimization phase), drug was started with a flexible dose of 25-75 mg/d tid or of 2 weeks (dose-optimization phase), drug was started with a flexible dose of 25-75 mg/d tid or bid and then reached a maximum dose of 450 mg/d, based on clinical response of the patient.

In the next 12 weeks (observation phase) the patient was given the drug and the average daily and weekly patient's seizures were recorded again. Patients were seen at treatment weeks of 2, 4, 8 and 12 and the rate of seizures and adverse events of drug were assessed. The outcome of this study was decrement of the patient's seizure frequency by 50%, compared to the baseline period. In flexible dose method, there is no requirement to increase the daily dose of patient’s drug to maximum dose of 450 mg/d, if fifty percent reduction in seizure frequencies can be achieved with lower doses of the drug.

**Patients**

Patients aged ≤18 years with a diagnosis of partial seizures as defined by the International League against Epilepsy (ILAE) Classification of Seizure (1981) were eligible for this study if the seizure was not adequately controlled by one to three AEDs administered as monotherapy or in combination before entering the study. All patients with a minimum of 4-6 partial seizures in the baseline phase of the study that never had a maximum of 28 free seizure days were included.

 Exclusion criteria included renal failure, current treatment with Vigabatrin or Felbamate, having absence seizure, status epilepticus, Lennox syndrome, myoclonic seizure, neurometabolic or progressive neurologic disorder.

**Statistical Analysis**

Efficacy analyses used an intent-to-treat population (n=29) that included all patients who received at least 1 dose of assigned treatment. Seizure rate during the treatment period was calculated on a last observation carried forward (LOCF) basis.

 The RRatio is defined as [(T-B)/(T+B)]-100, where B=Baseline Seizure Rate and T=Treatment Seizure Rate. The RRatio allows for a ‘‘symmetrized’’ percent change with a range of-100 to 100. Negative values of the RRatio represent an improvement in seizure rate and positive values indicate a worsening of seizure rate. Therefore the zero number shows no change in seizure frequency and -33 shows fifty percent reduction in seizure frequency. The main target of this study was RRatio of -33 in patients. The RRatio approaches normality, allowing for the use of parametric statistics, and facilitates seizure subtype analyses. For ease of clinical interpretation, a percentage change of seizure reduction was derived from the RRatio values as follows: ([200*RRatio]/[100-RRatio]). This parameter is more tangible for physicians because it specifically shows response rate and percent reduction in seizure frequency of patients. This analysis was performed using an analysis-of variance model with treatment as main effect and rank of the RRatio as the dependent variable.

 The major outcome variable was comparing fixed dose of pregabalin and the routine treatment. Secondary efficacy measures included percent changes, and changes from baseline were evaluated by Mann Whitney U test and Wilcoxon Signed Rank Test, comparing the difference to 0.

## Findings

Twenty nine children (15 boys and 14 girls) with age range of 1.3 to 17.5 years (mean: 8.35±0.89 years) with severe drug-resistant partial epilepsy were included in this study. There was positive family history of epilepsy in 51.7% of the cases; 20% had febrile seizure. The frequency of labor and prenatal problems was 55.2%; the most prevalent ones were asphyxia (5 cases), early rupture of amniotic sac (3 cases), intracerebral hemorrhage and meconium aspiration (each one 2 cases), hyperbilirubinemia, prematurity, neonatal seizure and TORCH insults (one case for each).

 79.3% of patients had symptomatic epilepsy and the rest was idiopathic. Patients reported using 1 (3.4%), 2 (34.5%), or ≥3 (62.1%) concomitant AEDs during the study. Six (20.7%) patients had a psychiatric disorder (ADHD, anxiety disorder, autism behavioral disorder, depression) simultaneously. The most common concomitant AEDs in respect to frequency were Valproic acid, Carbamazepine, Phenobarbital, and Topiramate. The initial dose of Pregabalin was 25-75 mg/d and maximum dose450 mg/d. The mean dose of the drug was 70 mg/d in the initiation and 225 mg/d at the end. 93.7% of patients had a complete exposure. Only 2 (6.3%) patients left the study because of unsatisfying results.

 The side effects were vertigo (10.3%), ataxia (6.9%), more than 7% weight gain (31%), drowsiness (24%) and blurred vision (3.4%). One case had drooling and one had agitation, which were mild to moderate and reduced gradually during treatment (Table 1).

**Table 1:** Frequency of pregabalin complications

**Complication**	**N0 (%)**
**Dizziness**	3 (10.3)
**Ataxia**	2 (6.9)
**Weight gain**	9 (31)
**Somnolence**	7 (24)
**Blurred vision**	1 (3.4)
**Others (drooling, irritability)**	2 (6.9)

The RRatio was -32.5, this indicates 36% reduction in seizures frequency. Responder rate (percent of patients with 50% reduction in seizure frequency) was 51.7%. Percent changes and changes from baseline was significant (*P*<0.0001). Distribution of responder rate between patients with positive and negative family history was equal (*P*<0.290); also no significant difference in the distribution of responder rate between symptomatic and idiopathic seizures was detected (*P*<0.511), but there was significant difference in distribution of this variable between genders (*P*<0.046).

## Discussion

Based on ILAE classification in 1981, generalized and partial seizures are two major types of epilepsy. Although there is good information about epilepsy, information about the various causes of refractory partial seizures is not enough^[^^14^^]^. Surgical treatment may be the treatment of choice in some patients, but the major axis of therapeutic plans is the use of different AEDs aimed at reducing seizure frequency in patients. In recent years the field of manufacturing AED has increased. This study indicated that daily administration of pregabalin is highly effective and well-tolerated as add-on therapy for controlling refractory partial seizures in children. With regard to other studies on efficacy of new anti-epileptic drugs, pregabalin has acquired the second place in performance and efficacy to help patients suffering from refractory partial seizures^[^^10^^]^. It should be noted that most of the new antiepileptic drugs are used as adjuvant or add-on therapy in treatment of drug resistant partial seizures. In this study, we also used pregabalin as add-on therapy in children with refractory partial seizures to reduce seizure frequency. Pregabalin has not a wide spectrum activity against different types of seizures and therefore should not be used for treatment of generalized seizures or Lennox-Gastaut syndrome^[^^14^^]^. The RRatio was -32.5 which indicates 36% reduction in frequency. As the responder rate shows, 51.7% of patients had 50% reduction of seizures. Carreno et al observed a responder rate of 52% and 39.6% at 6 and 12 months, respectively^[^^13^^]^. In other studies, the responder rate was 31.3% and 40% for a pregabalin flexible dose regimen^[^^2^^,^^8^^,^^15^^]^.

 As in current study, we used flexible dosing regimen to enhance tolerability of pregabalin as well as achieving efficacy to simulate general practice adjustment of dosage for each individual patient^[^^2^^]^. The treatment initiated with the effective starting dose of 25-75 mg/day followed by an opportunity for stepwise dose adjustment for those patients requiring increased doses to optimize efficacy and tolerability. The maximum dose was 450 mg/day.

 Pregabalin appears safe, the adverse effects are predominantly dose-related that are associated with the CNS complications such as dizziness, ataxia, and somnolence. Despite the relatively high frequency of adverse effects, particularly at higher doses, they infrequently result in discontinuation, and often are transient resolving in the first few weeks of treatment^[^^7^^,^^9^^,^^14^^,^^15^^]^ .

 On the other hand, adverse effects of anti-epileptic drugs are a particular concern in epileptic patients which have high frequency and strong association with poor health-related quality of life (HRQOL)^[^^16^^-^^18^^]^. Therefore, AEDs with a favorable side effect profile are of utmost importance to the optimal management of epilepsy particularly in an add-on setting as multidrug treatment increases the risk of toxicity. In the present study, most side effects were mild to moderate in intensity leading to discontinuation in none of patients. 

 Our research is limited by the short observation period and, most importantly, by the lack of a control group, which limits the extent to which the results can be generalized. ‘‘Real world’’ studies such as this provide useful insight into the use of pregabalin in typical clinical situations especially in children as we have lack of international pediatric data, for example there is no mention of a standard dose of this drug in children in academic resources.

## Conclusion

The results of this 12-week, open-label study extend the findings of previous studies to the local Iranian population, which suggest efficacy for pregabalin in the treatment of children with refractory partial seizures. This was evidenced by statistically significant reductions in seizure frequency in patients. Pregabalin was generally well tolerated and the safety profile was comparable to previously reported studies.
